# Supplementary material for: Phenolic Compounds and Antioxidant Capacity Comparison of Wild-Type and Yellow-Leaf gl1 Mutant of Lagerstroemia indica
Source: Plants (Basel). 2024 Jan 20;13(2):315. doi: 10.3390/plants13020315 (PMC10818265; doi:10.3390/plants13020315)
Supplement: Supplementary file 1 [file plants-13-00315-s001.zip › Supplementary material.pdf]

Table S1. The phenylpropanoid metabolism proteins with significant differentially down-regulated expression in the mutant *gl1* compared with WT.

Protein name	Describe	Accession No.	Unique Peptides	Mol. Weight [kDa]	log_2 (*gl1*/WT)
Dirigent	dirigent-like protein	PKI48616.1	6	21.36	-7.43
POD	peroxidase [EC:1.11.1.7]	OWM81649.1	8	32.92	-6.53
POD	peroxidase [EC:1.11.1.7]	XP_010031113.1	5	37.11	-4.56
POD	peroxidase [EC:1.11.1.7]	PKI44004.1	5	33.81	-4.29
POD	peroxidase [EC:1.11.1.7]	PKI72026.1	8	39.85	-1.81
POD	peroxidase [EC:1.11.1.7]	OWM83503.1	11	41.62	-1.67
POD	peroxidase [EC:1.11.1.7]	PKI72026.1	4	26.55	-1.32
COMT	caffeic acid 3-O-methyltransferase [EC:2.1.1.68]	XP_023919597.1	4	41.86	-5.16
COMT	caffeic acid 3-O-methyltransferase [EC:2.1.1.68]	AID68566.1	4	43.32	-1.43
CAD	cinnamyl alcohol dehydrogenase [EC:1.1.1.195]	OMO73994.1	3	39.02	-4.59
CAD	cinnamyl alcohol dehydrogenase [EC:1.1.1.195]	XP_018827699.1	2	39.01	-3.10
CAD	cinnamyl alcohol dehydrogenase [EC:1.1.1.195]	OWM64820.1	2	27.667	-1.93
CAD	cinnamyl alcohol dehydrogenase [EC:1.1.1.195]	PKI61458.1	2	38.67	-1.76
4-CL	4-coumarate:CoA ligase [EC:6.2.1.12]	PKI52297.1	3	38.20	-3.57
4-CL	4-coumarate:CoA ligase [EC:6.2.1.12]	OWM65790.1	2	23.36	-3.08
CCoAOMT	caffeoyl-CoA O-methyltransferase [EC:2.1.1.104]	XP_015869695.1	7	27.87	-2.57
HCT	shikimate O-hydroxycinnamoyl transferase [EC:2.3.1.133]	XP_010034043.2	2	43.75	-1.13

Table S2. The list of 193 differential metabolites including 84 up-regulated and 109 down-regulated ($p < 0.05$ and FC > 2 or FC < -2).

Please see the Supplementary File in EXCEL

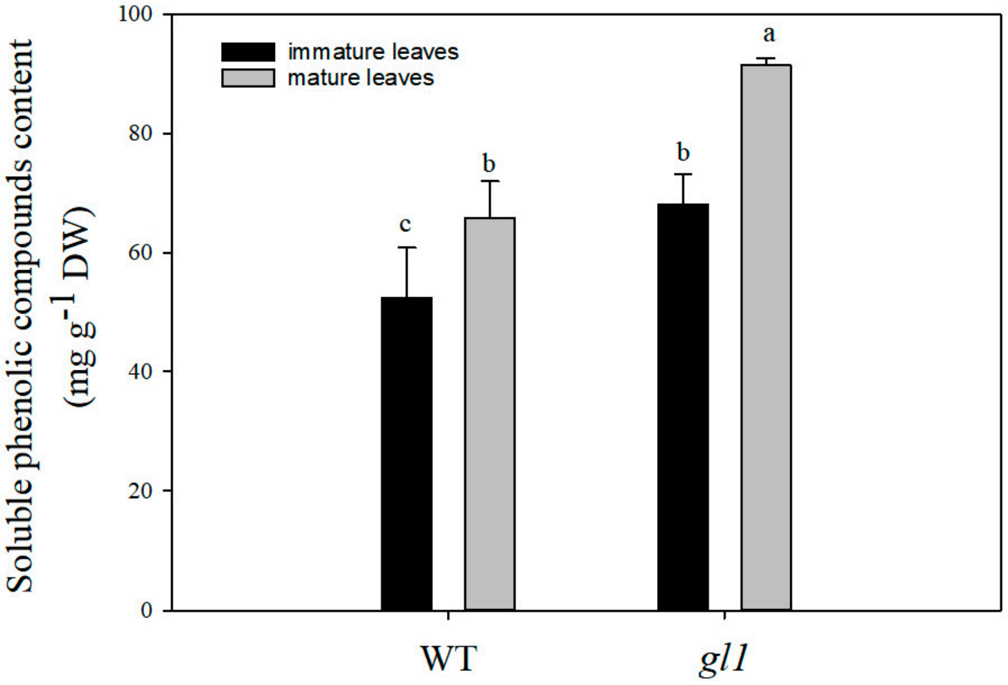

Figure S1. The contents of soluble phenolic compounds in the immature and mature leaves of the mutant *gl1* and WT. The vertical bars are presented as mean ± SD, n = 3. The different letters indicate significant differences at $p < 0.05$ level between WT and *gl1* mutant.

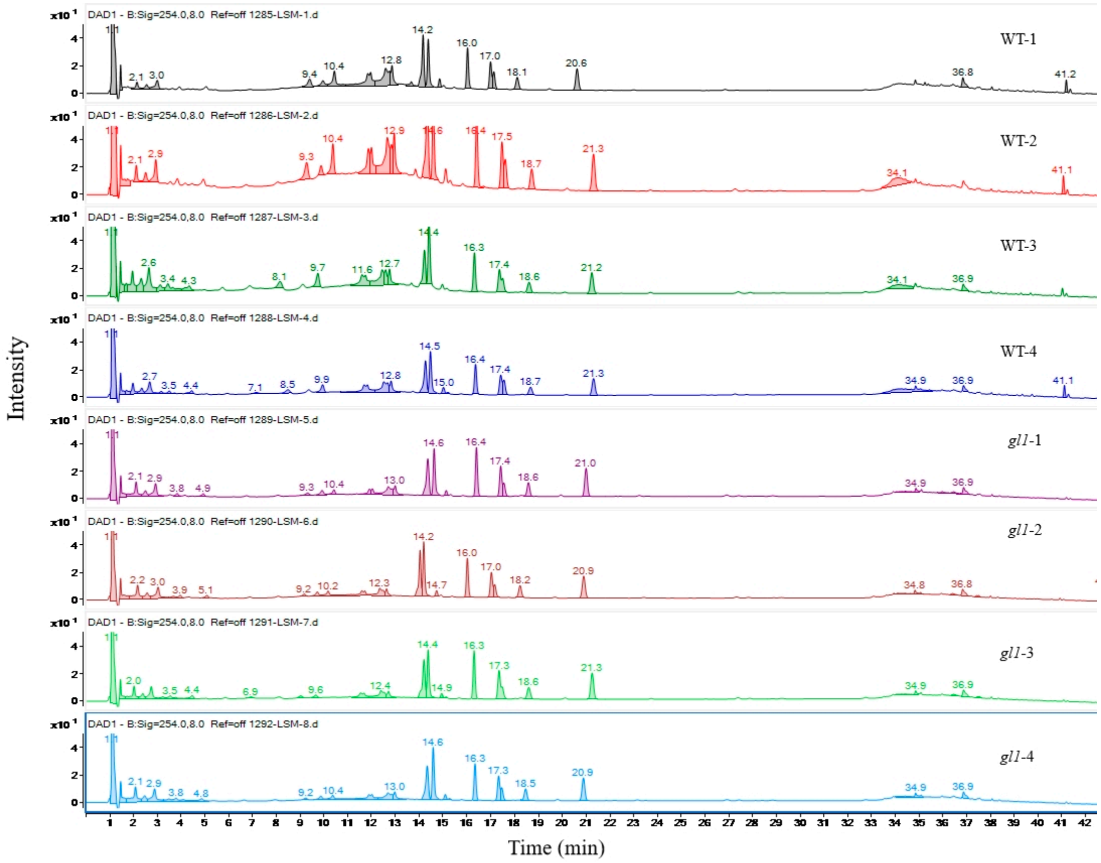

Figure S2. The total ion chromatogram of metabolites extract in WT and the *gl1* mutant.
